# Lifting the fog in intermediate-risk (submassive) PE: full dose, low dose, or no thrombolysis?

**DOI:** 10.12688/f1000research.17861.1

**Published:** 2019-03-25

**Authors:** Amyn Bhamani, Joanna Pepke-Zaba, Karen Sheares

**Affiliations:** 1 Department of Respiratory Medicine, Basildon and Thurrock University Hospital, Basildon, Essex, SS16 5NL; 2 Pulmonary Vascular Diseases Unit, Royal Papworth Hospital, Cambridge, CB23 3RE; 3 Department of Respiratory Medicine, Cambridge University Hospitals NHS Foundation Trust, Hills Road, Cambridge, CB2 0QQ

**Keywords:** Pulmonary embolism, intermediate risk, thrombolysis

## Abstract

Acute pulmonary embolism (PE) is a disease frequently encountered in clinical practice. While the management of haemodynamically stable, low risk patients with acute PE is well established, managing intermediate disease often presents a therapeutic dilemma. In this review, we discuss the various therapeutic options available in this patient group. This includes thrombolysis, surgical embolectomy and catheter directed techniques. We have also explored the role of specialist PE response teams in the management of such patients. ​

## Introduction

Acute pulmonary embolism (PE) is a relatively common disease with a variable clinical presentation. The annual incidence of diagnosed cases in the UK has previously been reported as 34.2 per 100,000 person-years ^1^, and it is estimated that there were 2300 deaths from the condition in 2012 ^2^. Data from the US suggest a far higher incidence. The Centers for Disease Control and Prevention estimates that there are 60,000 to 100,000 annual deaths from deep vein thrombosis or PEs in the US, and sudden death is felt to be the first symptom in about a quarter of cases ^3^. In Europe, epidemiological models based on data from six European Union countries have estimated the total number of PE events per annum as 95 per 100,000 ^4^.

An important challenge in the management of acute PE is patient risk stratification. Massive or high-risk PE is characterised by sustained hypotension (systolic blood pressure of less than 90 mm Hg for at least 15 minutes or requiring inotropic support) not attributable to another cause. In contrast, submassive or intermediate-risk PE refers to an acute PE without systemic hypotension but with either right ventricular (RV) dysfunction or myocardial necrosis ^5^. This distinction is important as massive PE is associated with increased mortality. In the International Cooperative Pulmonary Embolism Registry (ICOPER), the 90-day mortality rate for patients with acute PE and systolic blood pressure of less than 90 mm Hg at presentation was 52.4% compared with 14.7% in the remainder of the cohort ^6^. Additionally, in the Management Strategy and Prognosis of Pulmonary Embolism Registry of 1001 patients with acute PE, in-hospital mortality was 8.1% for haemodynamically stable patients versus 25% for those presenting with cardiogenic shock and 65% for those requiring cardiopulmonary resuscitation ^7^.

Low-molecular-weight heparin is a well-established initial therapy for patients with acute PE who remain haemodynamically stable. It has been found to reduce the incidence of complications and thrombus size compared with unfractionated heparin ^8^, and its relative ease of administration is an added advantage. Typically, such patients subsequently receive parenteral anticoagulants followed by vitamin K antagonists or the newer direct oral anticoagulant (DOAC) agents.

## Full-dose thrombolysis

Over the past 50 years, almost 20 randomised studies on thrombolytic therapy in acute PE have been published and their results reviewed in meta-analyses ^9– 12^. The severity of PE, the type of thrombolytic agent and heparin used, and the dose and duration of administration varied across the trials. Furthermore, the clinical criteria defining haemodynamic instability, shock and haemorrhage were not standardised. Only a few studies focused on clinical outcomes, and most lacked the statistical power to permit definite conclusions.

Major haemorrhage following thrombolytic therapy also remains a concern, and the reported incidence is between 0 and 33% depending on the type and dose of thrombolytic agent used ^13^. The ideal dose of thrombolysis and how it should be administered are also unclear.

International guidelines recommend thrombolysis for the management of PE patients with haemodynamic instability ^14– 16^. However, the role of thrombolysis in the management of haemodynamically stable patients with submassive or intermediate-risk disease is still a matter of debate ^17^.

The large, randomised Pulmonary Embolism Thrombolysis (PEITHO) trial compared the composite end point of mortality and haemodynamic collapse in over 1000 intermediate-risk patients who received heparin plus tenecteplase or placebo. Tenecteplase significantly reduced the risk of haemodynamic decompensation within 7 days but also was associated with a 10-fold increase in intracranial haemorrhage (2% versus 0.2%) and a fivefold increase in major haemorrhage (6.3% versus 1.2%). The 2% rate of intracranial haemorrhage may reflect a combination of full-dose tenecteplase plus a simultaneous loading bolus of heparin. Surprisingly, the follow-up results of PEITHO showed that thrombolysis did not affect long-term survival or reduce residual dyspnoea, RV dysfunction, or the incidence of chronic thromboembolic pulmonary hypertension (CTEPH) ^18^.

The results of various meta-analyses comparing full-dose thrombolysis with standard anticoagulation have been mixed. Chatterjee *et al*. compared outcomes in patients with acute PE treated with thrombolysis compared with anticoagulation alone ^19^. This included patients with intermediate-risk disease. The use of thrombolysis was associated with lower all-cause mortality and risk of recurrent PE. However, the risk of intracranial haemorrhage was also greater in thrombolysed patients ^19^.

Conversely, Wan *et al*. found no evidence for a benefit of thrombolytic therapy compared with heparin for the initial treatment of unselected patients with acute PE ^20^. However, there was a suggested benefit for haemodynamically unstable patients which should be weighed up against the statistically significant increased risk of non-major bleeding ^20^. Similarly, Dong *et al*. concluded that outcomes in terms of death rate, recurrent PE and haemorrhagic events were similar in patients who received thrombolytic therapy compared with placebo or heparin ^21^.

The development of alternative therapeutic modalities for patients presenting with large-volume PE, with or without haemodynamic compromise, has been the subject of great interest. Whereas Jimenez *et al*. concluded that recanalisation procedures (full-dose, low-dose and catheter-assisted thrombolysis) did not offer a clear advantage in the treatment of PE compared with standard anticoagulation alone ^22^, a number of novel therapeutic modalities have recently been proposed. However, their precise role in clinical practice remains unclear. We have therefore attempted to review some of these and analysed available data relating to their use in a clinical setting. We have focused in particular on submassive or intermediate-risk disease.

## Low-dose thrombolysis

Low-dose thrombolysis has been suggested as a potential treatment strategy for acute PE, particularly in patients presenting with intermediate-risk disease. It remains unclear whether early thrombolysis in this patient group has an impact on clinical symptoms, functional limitation, or CTEPH at long-term follow-up.

A small randomised trial of 83 patients suggested that thrombolysis, compared with anticoagulation alone, might improve functional capacity at 3 months. In the PEITHO trial, long-term (at 41.6 ± 15.7 months) clinical follow-up was available for 358 patients with intermediate-risk PE who survived the acute phase; persisting symptoms, mainly dyspnoea, were present in 33% of the patients. However, the degree of functional limitation was mild in the majority of cases regardless of whether the patients had been randomly assigned to thrombolysis or anticoagulation alone. In agreement with the clinical findings, the majority of patients (85% in the tenecteplase arm and 96% in the placebo arm) had a low or intermediate probability—based on the definition of the European Society of Cardiology (ESC) guidelines—of persisting or new-onset PH at echocardiographic follow-up. A standardised diagnostic work-up for CTEPH was not mandated by the trial protocol. Consequently, the findings of this study do not support a role for thrombolysis with the aim of preventing long-term sequelae after intermediate-risk PE, although they are limited by the fact that clinical follow-up was available for only 62% of the study population.

The Moderate Pulmonary Embolism Treated with Thrombolysis (MOPETT) trial evaluated the role of half-dose (0.5 mg/kg up to a maximal dose of 50 mg) thrombolysis with tissue plasminogen activator (tPA) and anticoagulation versus standard anticoagulation alone in the management of patients with moderate PE. A statistically significant reduction in the development of pulmonary hypertension, defined in the trial as pulmonary artery systolic pressure (PASP) of greater than 40 mm Hg by echocardiography, was reported in the treatment group compared with the control group. There was no significant difference in mortality or bleeding, although the average length of hospital admission was shorter in the treatment group ^23^. However, it must be noted that the definition of pulmonary hypertension in the trial does not meet internationally defined diagnostic criteria for this condition. Nevertheless, patients in the treatment arm were found to have a greater reduction in PASP compared with the control group, and although long-term functional outcomes were not formally assessed, these results may be significant in this regard.

The efficacy of low-dose thrombolysis compared with full-dose has been evaluated by Wang *et al*., who demonstrated similar improvements in RV dysfunction and radiological clearance in patients who received half-dose tPA (50 mg/2 hours) compared with those who received the full dose (100 mg/2 hours) ^24^. Bleeding was less common in patients who received the lower-dose regimen, although this difference was not statistically significant ^24^. Similarly, Goldhaber *et al*. found no differences between a reduced-dose bolus and full-dose tPA with respect to bleeding complications. Additionally, efficacy was similar in the two treatment groups ^25^.

The role of low-dose thrombolysis in the management of post-operative patients has also been considered. Recent surgical intervention has generally been accepted as a relative contraindication for thrombolysis, particularly for those who have undergone cardiothoracic procedures. Shen *et al*. described the successful use of low-dose thrombolysis with 25 mg tPA in a high-risk patient who had a cardiac arrest soon after lung resection surgery ^26^. The patient was still alive after 6 months despite developing a mediastinal haematoma that required surgical drainage and blood transfusions ^26^. Low-dose thrombolysis has also been successfully used in the treatment of a patient with submassive PE and right heart thrombus following cardiac surgery ^27^.

## No thrombolysis

### Surgical embolectomy

First successfully performed by Kirschner in 1924, surgical embolectomy fell out of favour following the advent of thrombolytic therapy in the 1970s. However, the risk of bleeding associated with systemic thrombolysis has meant that this modality has re-emerged as a treatment of choice for patients in whom systemic thrombolysis is contraindicated. According to the ESC guidelines, surgical embolectomy should be considered (where surgical expertise and resources are available) in such patients and in those in whom thrombolysis has been unsuccessful in achieving haemodynamic stability. This procedure may also be a consideration in patients with acute PE who require surgical excision of the right atrial thrombus or closure of a patent foramen ovale to prevent paradoxical emboli.

Results from small retrospective analyses have previously shown that surgical embolectomy is an effective treatment modality with 30-day mortality figures of 6 to 8%. This includes critically unwell patients presenting with cardiogenic shock and cardiorespiratory arrest requiring cardiopulmonary resuscitation ^28, 29^. More recently, a multi-centre analysis of over 200 patients by Keeling *et al*. quoted in-hospital mortality of 11.7% ^30^. This included patients who experienced pre-operative cardiac arrest ^30^.

Table 1 presents a comparison of mortality rates following surgical embolectomy ^28– 35^.

**Table 1.** Comparison of mortality rates following surgical embolectomy.

Study	Number of centres	Number of patients	Type of patients	Mortality
Lee *et al*. ^34^ (2018)	Multi-centre (New York state)	257	Not specified	13.2% 30-day mortality 23.9% 5-year mortality
Pasrija *et al*. ^31^ (2018)	Single-centre	55	Massive and submassive	7% in-hospital mortality 9% 1-year mortality
Lehnert *et al*. ^35^ (2017)	Single-centre	41	High- and intermediate-risk	High risk: 14% 30-day mortality and 32% 5-year mortality Intermediate risk: 23% 5-year mortality
Keeling *et al*. ^30^ (2016)	Multi-centre (four centres)	214	Massive and submassive	11.7% in-hospital mortality
Aymard *et al*. ^32^ (2013)	Single-centre	28	Massive	17% long-term (over mean 63 ± 21-month follow-up) mortality
Wu *et al*. ^33^ (2013)	Single-centre	25	High-risk	20% in-hospital mortality
Kadner *et al*. ^29^ (2008)	Single-centre	25	Central and paracentral pulmonary embolism	8% 30-day mortality
Leacche *et al*. ^28^ (2005)	Single-centre	47	Massive	14% 1-year mortality 17% 3-year mortality

The role of surgical embolectomy in the management of patients in whom thrombolysis is not contraindicated is more controversial, particularly because of the paucity of head-to-head trials comparing the two treatment modalities. Nevertheless, recently published data suggest that short- and long-term mortality rates from the two interventions are comparable ^34^. Furthermore, surgical embolectomy has been associated with similar intracranial bleeding rates but fewer major bleeding complications compared with thrombolysis.

Surgical embolectomy has also been shown to result in a greater difference between pre- and early post-operative systolic pulmonary artery pressure and RV diameter in patients presenting with acute high-risk PE compared with thrombolysis ^36^. Additionally, Lehnert *et al*. demonstrated that surgical embolectomy resulted in a significantly lower amount of residual emboli compared with thrombolysis ^35^.

## Catheter-directed techniques

The growing role of percutaneous interventions in modern medicine has led to an interest in the development of similar strategies for the treatment of PE, especially in patients in whom thrombolysis is contraindicated or not indicated. Various modalities of catheter-directed mechanical thrombectomy and localised low-dose thrombolysis have been developed over the past few years. Although data related to their use are limited at present, it is expected that the next few years will see further progress in this regard.

The Pulmonary Embolism Response to Fragmentation, Embolectomy, and Catheter Thrombolysis (PERFECT) data study assessed clinical outcomes using changes in mean pulmonary artery pressure and right heart strain in 101 patients who underwent catheter-directed or pharmaco-mechanical thrombectomy and/or catheter-directed thrombolysis for massive or submassive PE. Data from the study suggested improved clinical outcomes in these patients. Additionally, the average dose of thrombolysis was lower than that used for systemic therapy and there were no major bleeding complications ^37^.

### Ultrasound-assisted catheter-directed thrombolysis

The addition of ultrasound assistance to localised catheter-directed thrombolysis is postulated to facilitate the delivery of the thrombolytic agent to the intended target and additionally accelerate fibrinolysis by causing disruption of uncrosslinked fibrin fibres into smaller fibres ^38^. However, a retrospective comparison of ultrasound-assisted and standard catheter-directed thombolysis by Liang *et al*. revealed no significant difference in outcomes or complication rates between the two techniques ^39^. More recently, similar results were reported by Schissler *et al*., who found no significant differences in length of hospital admission, RV dysfunction on follow-up echocardiography and 1-year mortality in patients who received this treatment modality compared with standard anticoagulation alone ^40^. The SEATTLE II trial is thus far the largest study looking at the efficacy and safety of ultrasound-assisted catheter-directed fibrinolysis. One hundred fifty patients with either massive or submassive PE were given 24 mg of tPA, administered by either unilateral or bilateral catheters. The change in RV-to-left ventricle (LV) diameter ratio was used as the primary efficacy outcome. The trial showed a mean reduction of 0.42 along with a statistically significant reduction in mean pulmonary artery systolic pressure. Although one patient had a severe bleed in the form of a groin haematoma resulting in hypotension, there were no intracranial bleeds ^41^. It is worth noting, however, that this was a single-arm trial which involved no head-to-head comparison with other therapeutic modalities. Similar physiological improvements were noted by Kaymaz *et al*., who assessed outcomes in 141 patients who received this modality ^42^. Interestingly, bleeding rates and long- and short-term mortality were not related to age ^42^.

A randomised controlled trial comparison of additional ultrasound-assisted catheter-directed thrombolysis with anticoagulation versus anticoagulation alone was carried out by Kucher *et al*. ^43^. The addition of ultrasound assistance resulted in a significant reduction in RV dilatation compared with anticoagulation alone. No increased risk of bleeding was demonstrated ^43^. A trial comparing the safety and efficacy of this modality with peripheral, low-dose thrombolysis is ongoing ^44^.

Recruitment into another trial comparing standard and ultrasound-assisted catheters is already under way, and both short- and long-term outcomes are being compared ^45^. Additionally, the Optimum Duration of Acoustic Pulse Thrombolysis Procedure in Acute Pulmonary Embolism (OPTALYSE PE) study is attempting to determine the ideal dose of thrombolytic agent and length of ultrasound procedure in patients with submassive PE ^46^.

## Mechanical embolectomy

Like catheter-directed therapy, mechanical embolectomy is used relatively infrequently in clinical practice and data regarding its use have historically been limited to isolated case reports and cohort studies involving small numbers of patients. This treatment modality was recently the subject of the multi-centre FlowTriever Pulmonary Embolectomy (FLARE) Clinical Study trial, which assessed the use of an aspiration catheter in 106 patients with intermediate-risk PE ^47^. Preliminary results suggest a significant reduction in RV size and a relatively low rate of major adverse events ^48^. However, in the absence of a control group, it is difficult to draw any definitive conclusions regarding its long-term feasibility outside a trial setting. Nevertheless, the FlowTriever system recently received US Food and Drug Administration approval for the treatment of PE in the US and this may lead to a more widespread use of this technology in the future.

Thrombus fragmentation with the injection of saline at high velocity, so-called rheolytic embolectomy, is another form of mechanical embolectomy that has been used in the management of acute PE. The smaller fragments can then be suctioned out of the vessel with a catheter. This technique has been described in various case reports, including in an especially challenging case involving a patient with heparin-induced thrombocytopenia and a recent ischaemic stroke who subsequently presented with an acute PE ^49^. However, data from small cohort studies have not been as encouraging. In a meta-analysis of various modern catheter-directed thrombolysis modalities, Kuo *et al*. found that this technique was associated with the highest complication rates, including five deaths in the total cohort of 68 ^50^. Additionally, although Zeni *et al*. reported good immediate angiographic improvement following the use of rheolytic embolectomy in 17 patients, 10 subsequently required an adjuvant thrombolytic infusion ^51^.

The use of a rotational catheter to cause thrombus fragmentation and at least partial clearance of a central embolic occlusion has also been described. This technique can be combined with localised infusion of a thrombolytic agent to improve its effectiveness ^52^. However, despite approval for use of a rotational thrombectomy system in the treatment of peripheral and arteriovenous dialysis fistulae thrombosis in the US, data regarding its role in the management of patients with pulmonary emboli are lacking.

## Pulmonary embolism response teams

In 2012, Massachusetts General Hospital introduced the pulmonary embolism response team (PERT). The team is composed of specialists from a number of clinical backgrounds and provides expert multi-disciplinary evaluation and management input for intermediate- and high-risk patients with PE ^53^. Although initial data suggested that the most common treatment recommended by the team was anticoagulation alone, 11% of patients received systemic or catheter-directed thrombolysis. Additionally, PERT activations increased by 16% every 6 months over the course of the initial 30-month period, highlighting the potentially greater role that such teams are likely to play in the management of acute PE in the future ^54^. The argument for the use of such teams is strengthened by a recent analysis of outcomes for patients treated by activation of the PERT pathway in Kentucky. This showed significantly lower intensive care unit and overall in-hospital length of stay compared with patients treated at the discretion of the attending team alone. However, there was no statistically significant difference in mortality between the two groups ^55^.

A number of other PERT programmes have subsequently been developed in the US and led to the development of the PERT Consortium, which intends to be “the driving force behind increased survival rates and the future of PE treatment” ^56^.

## Conclusions

The optimum management of acute PE continues to be a clinical challenge. The early management of intermediate-risk disease, in particular, remains a subject of debate. Although anticoagulation continues to be the mainstay of treatment, head-to-head trials of reperfusion strategies are still needed. PE referral centres and PERTs will facilitate multi-disciplinary decision-making for treatment of higher-risk patients and hopefully improve patient outcomes.
